# E2F2 and CREB cooperatively regulate transcriptional activity of cell cycle genes

**DOI:** 10.1093/nar/gkt821

**Published:** 2013-09-12

**Authors:** Usua Laresgoiti, Aintzane Apraiz, Miguel Olea, Jone Mitxelena, Nerea Osinalde, José A. Rodriguez, Asier Fullaondo, Ana M. Zubiaga

**Affiliations:** ^1^Department of Genetics, Physical Anthropology and Animal Physiology, University of the Basque Country, UPV/EHU, Bilbao 48940, Spain and ^2^Department of Biochemistry and Molecular Biology, University of the Basque Country, UPV/EHU, Bilbao 48940, Spain

## Abstract

E2F2 is essential for the maintenance of T lymphocyte quiescence. To identify the full set of E2F2 target genes, and to gain further understanding of the role of E2F2 in transcriptional regulation, we have performed ChIP-chip analyses across the genome of lymph node–derived T lymphocytes. Here we show that during quiescence, E2F2 binds the promoters of a large number of genes involved in DNA metabolism and cell cycle regulation, concomitant with their transcriptional silencing. A comparison of ChIP-chip data with expression profiling data on resting *E2f2^−^^/^^−^* T lymphocytes identified a subset of 51 E2F2-specific target genes, most of which are upregulated on E2F2 loss. Luciferase reporter assays showed a retinoblastoma-independent role for E2F2 in the negative regulation of these target genes. Importantly, we show that the DNA binding activity of the transcription factor CREB contributes to E2F2-mediated repression of *Mcm5* and *Chk1* promoters. siRNA-mediated CREB knockdown, expression of a dominant negative KCREB mutant or disruption of CREB binding by mutating a CRE motif on *Mcm5* promoter, relieved E2F2-mediated transcriptional repression. Taken together, our data uncover a new regulatory mechanism for E2F-mediated transcriptional control, whereby E2F2 and CREB cooperate in the transcriptional repression of a subset of E2F2 target genes.

## INTRODUCTION

Mammalian E2F is a family of transcription factors, originally discovered for their crucial role in the control of cell cycle progression through the activation or suppression of a group of responsive genes (1,2). Traditionally, the mammalian E2F family has been divided into ‘activators’ (E2F1–3) and ‘repressors’ (E2F4–8). However, recent *in vivo* data have challenged this oversimplified classification. Indeed, accumulating evidence suggests that most E2Fs can function both as activators as well as repressors of target genes, including those coding for microRNAs (3–8). However, the mechanisms underlying this bimodal impact of individual E2Fs remain to be elucidated.

Characterization of mouse models lacking individual E2Fs has revealed that these factors play unique roles in development, tissue homeostasis and tumor formation (9,10). Specific regulation of different sets of target genes by individual E2Fs may account for the phenotypes observed in the absence of these proteins. In this regard, chromatin immunoprecipitation assays coupled to DNA microarray analysis (ChIP-chip) or to whole-genome sequencing technology (ChIP-Seq), and microarray expression profiling analyses in cells overexpressing individual E2Fs, have revealed that E2F factors not only regulate genes necessary for G1/S transition but also a wide spectrum of genes with diverse biological functions, including regulation of apoptosis, autophagy, mitosis, chromosome organization, macromolecule metabolism or differentiation (2,10).

The transcriptional activity of E2F1–5 is controlled primarily by their temporal association with the retinoblastoma (RB) family of tumor suppressor proteins (1,2), whereby interaction of E2Fs with RB at the promoter of target genes leads to transcriptional repression. However, there is also evidence for RB-independent transcriptional repression mediated by E2F1–5 factors (11–13), although the mechanisms for this type of repression are largely unknown. E2F target specificity is thought to arise from unique interactions between E2Fs and other transcription factors at a particular promoter (14). Some of these E2F-interacting transcription factors, such as SP1, p110 CUX1, ALY and RYBP, have already been identified during the past few years (15–18). However, given the complexity of the transcriptional network regulated by the E2F family, the list of proteins that contributes to E2F promoter specificity is likely to include other, yet to be identified, transcription factors.

A better understanding of the role of E2F transcription factors *in vivo* requires the identification of the full range of genes regulated by each individual E2F. Our group and others have reported that *E2f2^−^^/^^−^* and compound *E2f1^−^^/^^−^/E2f2^−^^/^^−^* mice exhibit defects in the proliferative properties of hematopoietic cells (19–22), and transcriptomic analyses have linked hyperproliferation of *E2f2^−^^/^^−^* lymphocytes to deregulation of a large set of E2F target genes, particularly in quiescence (4). Whether E2F2 is directly involved in the transcriptional regulation of these genes has not yet been clarified.

In this work, we have performed a genome-wide search for E2F2 binding sites by ChIP-chip in quiescent T lymphocytes. We have identified a large collection of E2F2-bound genes involved in DNA replication/repair and cell cycle control. Gene expression profiling data from E2F2-deficient T lymphocytes indicate that a subset of these genes, including *Mcm5* and *Chk1*, is uniquely repressed by E2F2. Importantly, we demonstrate that CREB binding to *Mcm5* and *Chk1* promoters facilitates E2F2-mediated transcriptional repression of these genes. These results suggest a functional interaction between E2F2 and CREB on the promoters of E2F target genes, thereby unveiling a novel aspect of E2F2-mediated transcriptional regulation.

## MATERIALS AND METHODS

### Mice and culture conditions

*E2f2^−^^/^^−^* and wild-type (WT) mice (C57Bl6:129Sv background) were maintained on a normal light/dark cycle in cages with microisolator lids, and were genotyped by standard polymerase chain reaction (PCR) technology, as previously described (4). All procedures were approved by the Animal Care and Use Committee of the University of the Basque Country.

T cell preparation and culture were carried out in complete medium (RPMI 1640 supplemented with 10% FBS, 2 mM l-glutamine, 50 U/ml penicillin and 50 µg/ml streptomycin). Lymph nodes were extracted from 4-week-old WT and *E2f2^−^^/^^−^* mice and mechanically dissociated between two pieces of ground glass. Debris was allowed to settle, and the cells were collected. For analysis of T cell receptor-mediated responses, T lymphocytes (1.7 × 10^6^/ml) were stimulated for the indicated times with immobilized antibodies against CD3 (145.2C11, BD, 30 µg/ml). Human embryonic kidney (HEK) 293 T cells and human U2OS osteosarcoma cells were maintained in Dulbecco’s modified Eagle’s medium supplemented with 10% FBS.

For cell synchronization at mitosis, U2OS cultures were incubated with thymidine (2 mM) for 18 h. Subsequently, cells were washed and cultured for an additional 20 h in fresh medium. Nocodazole (100 ng/ml) was added to the cultures for the last 16 h. Cells at M phase were collected by shaking off the plates and seeded in complete medium for subsequent analyses. To assess cell cycle distribution, cells were fixed with chilled 70% ethanol, treated with RNAse, stained with 50 µg/ml propidium iodide and analyzed by flow cytometry (FACSCalibur, BD). Cell cycle distribution analysis was performed with ModFit LT software, and data were represented with WinMDI2.8 software.

### Plasmid description

Plasmid constructs pChk1-Luc (encompassing the −613 to +1664 genomic region of human Chk1), pE2F-Luc (containing three canonical E2F motifs), pCRE-Luc (containing four canonical CRE motifs), pRc-CMV-HA-E2F2 and pCREB-VP16 have been described previously (23–26). Plasmids pCMV-CREB and pCMV-KCREB were purchased from Clontech. To construct the pMcm5-Luc reporter plasmid, a 659 bp fragment (−528 to +131) of the murine *Mcm5* genomic region was cloned into the pCR2.1-TOPO vector using the ‘TOPO TA cloning’ kit (Invitrogen). After digestion with MluI and BglII, the generated insert was cloned into the pGL3-basic luciferase reporter vector (Promega).

Site-directed mutagenesis of the CRE motif in p*Mcm5-Luc*, and E2F motifs in *pMcm5-Luc* and in *pChk1-Luc* reporter plasmids was carried out using either the QuikChange Lightning Site-Directed Mutagenesis Kit or the QuikChange Lightning Multi Site-Directed Mutagenesis Kit (Stratagene), following manufacturer’s directions (See Supplementary Table S1 for nucleotide sequences of the primer sets used for mutagenesis).

DNA fragments encoding HA-tagged E2F2 deletion mutants lacking the transactivation domain (HA-E2F2-ΔTRD) or the DNA binding domain (HA-E2F2-ΔDBD) were amplified by PCR using the pRc-CMV-HA-E2F2 vector as template. To amplify the fragment encoding HA-E2F2-ΔTRD (corresponding to amino acids 1–358), the forward primer (5′-CTGCAAGGGCCCCGGGCCT-3′) in combination with the reverse primer (5′-CTGCTGGGGGGTTGGCGCTGGT-3′) were used. To amplify the fragment encoding HA-E2F2(ΔDBD) (corresponding to amino acids 196–437), the forward primer (5′-TTTGAAGACCCCACCA-3′) and the reverse primer (5′-ATTAATCAACAGGTCCCCAAGG-3′) were used. PCR products were digested with BamHI and HindIII and ligated into the pEYFP-C1 vector (Clontech) to generate the corresponding pEYFP-HA-E2F2 deletion mutant. To create pEYFP-HA-E2F2-ΔDD (lacking the dimerization domain encompassing amino acids 197–358), pUC57-HA-E2F2-ΔDD was obtained (Genscript), and the insert was subcloned into the pEYFP-C1 vector using the BamHI/HindIII restriction sites. The orientation and integrity of all constructions were confirmed by DNA sequencing.

### Transfections, siRNA-mediated knockdown and luciferase activity assays

Transient transfections were performed by the calcium phosphate method in HEK293T cells, or with Fugene (Promega) in U2OS cells.

For knockdown of endogenous E2F2 and CREB, siRNA oligonucleotides were transfected at a final concentration of 10 nM using Lipofectamine RNAiMAX (Life Technologies) following the manufacturer’s recommendation.

For luciferase activity assays, cells at 40% confluency in six-well plates were transfected with 200 ng of the firefly luciferase reporter vector, 20 ng of the *Renilla* luciferase reporter vector (pRL-TK) and empty vector to 2 µg of DNA per transfection. Using the Dual-Luciferase Reporter Assay System (Promega), the reporter firefly luciferase activity was measured 48 h after transfection and was normalized to the transfection efficiency estimated by the activity of *Renilla* luciferase in each sample. Results were calculated as fold induction over the luciferase expression in cells that were transfected only with the appropriate reporter vector.

### Gene expression analyses

RNA extraction was performed with TRIzol Reagent (Life Technologies). RNA was purified with the RNeasy Mini Kit (Qiagen) and reverse transcribed into cDNA with the High-Capacity cDNA RT Kit (AB). Quantitative real-time PCR was performed as described previously (4). Sequences of PCR primers are listed in Supplementary Table S2.

For western blot analyses, cells were lysed in buffer containing 10 mM NaPO_4_H, pH 7.2; 1 mM EDTA; 1 mM EGTA; 150 mM NaCl; 1% NP-40 and a cocktail of protease and phosphatase inhibitors (Roche). Protein concentrations in supernatants were determined using a commercially available kit (DC Protein Assay from Bio-Rad). Twenty to fifty micrograms of protein were loaded per lane, fractionated in 10–12% sodium dodecyl sulphate-polyacrylamide gels and transferred onto nitrocellulose membranes (Bio-Rad). Antibodies against the following proteins were used: CREB (sc-186, Santa Cruz), p-CREB (sc-7978-R, Santa Cruz), E2F2 (sc-633, Santa Cruz) and β-Actin (A5441, Sigma). Immunocomplexes were visualized with horseradish peroxidase-conjugated anti-mouse or anti-rabbit IgG antibodies (Amersham), followed by chemiluminiscence detection (ECL, Amersham) with a ChemiDoc camera (Bio-Rad).

### Chromatin immunoprecipitation and ChIP-chip analysis

Chromatin immunoprecipitations and the quantification of immunoprecipitate-enriched DNA sequences by real-time PCR were performed as described previously (4). Sequences of PCR primers are listed in Supplementary Table S3. Antibodies used were E2F2 (sc-633, Santa Cruz), CREB (06-863, Millipore) and SV40Tag (sc-147, Santa Cruz).

In ChIP-chip experiments, DNA obtained by standard ChIP experiments together with 20 ng of total input DNA was amplified by ligation mediated-polymerase chain reaction (LM-PCR), following manufacturer’s recommendation (Roche NimbleGen). Labeling, hybridization and detection of protein binding sites was performed by NimbleGen Systems Inc. Briefly, ChIP samples and total input DNA were labeled with Cy5 and Cy3, respectively, and co-hybridized to the Mouse ChIP-chip 385 K RefSeq Promoter Array, 05545021001, a microarray containing the promoters of 19 000 mouse genes (each promoter encompassing a DNA sequence from −2000 to +500 bp). Data were extracted and peaks were detected with the NimbleScan Program, according to standard operating procedures by NimbleGen Systems Inc. This program also calculates False Discovery Rate (FDR) values for each peak.

Identification of overrepresented motifs in peaks detected by ChIP-chip was performed using the cis-regulatory element annotation system (CEAS) server at http://ceas.cbi.pku.edu.cn (27). The localization of E2F and CRE motifs in E2F2-bound promoters was carried out with the MotifLocator tool of the TOUCAN program (28). Cutoffs of 0.85 and 0.9 and the ‘Mouse 1 Kb Proximal 1000 ENSMUSG (3)’ background were used. The ModuleSearcher tool of the TOUCAN program was used to analyze promoter sequences in search of E2F and CRE motifs separated by <500 bp. Functional annotations were performed using DAVID at http://david.abcc.ncifcrf.gov/ (29). The parameter used in this study was Gene Ontology Biological Process term, level 5. Only categories of genes showing statistically significant (*P* < 0.05) enrichment over the background category lists (which include all the genes in the microarray) were considered.

### Statistical analysis

Data are presented as mean ± SD. The significance of the difference between two groups was assessed using the Student two-tailed *t*-test. A *P* < 0.05 was considered statistically significant.

## RESULTS

### Genome-wide chromatin immunoprecipitation analysis reveals E2F2 occupancy of target promoters during T lymphocyte quiescence

We have previously reported that E2F2 is essential for the maintenance of lymphocyte quiescence (4). To further explore the role of E2F2 in transcriptional regulation during quiescence, we made use of genome location analyses and expression profiling data to identify genes directly regulated by E2F2 during this stage. To assess the extent of promoter occupancy by E2F2 at a genome-wide level, we performed chromatin immunoprecipitation followed by microarray hybridization (ChIP-chip) assays in lymph node-derived primary lymphocytes (*n* = 4 mice per experiment; three independent experiments). After ChIP with an anti-E2F2 antibody, we prepared amplicons by LM-PCR, and probed promoter microarrays representing 19 000 mouse genes. As a negative control, we carried out parallel ChIP-chip experiments with an irrelevant anti-SV40TAg antibody (anti-T). Using the NimbleScan program, we selected those E2F2-binding sites identified in at least two of the three immunoprecipitations performed with anti-E2F2 antibody. A total of 839 genes (Supplementary Table S4) were found to be selectively enriched with anti-E2F2 antibodies (FDR < 0.05).

**Figure 1.**
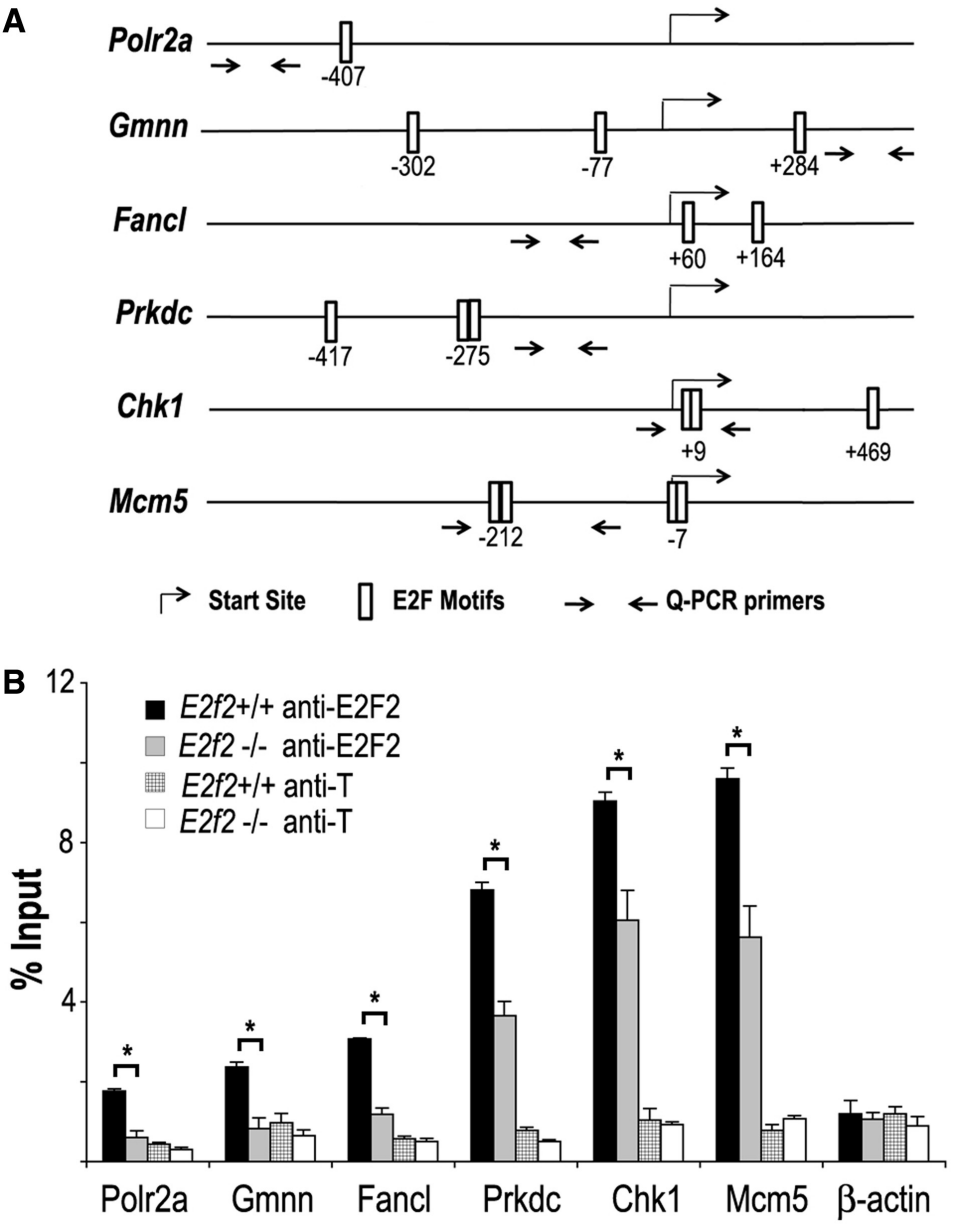
E2F2 is recruited to the promoter region of E2F target genes during quiescence. (**A**) Schematic representation of mouse *Chk1*, *Mcm5*, *Prkdc*, *Gmnn*, *Polr2a* and *Fancl* promoter regions, indicating the localization of consensus E2F motifs detected with *MotifLocator* at a 0.9 threshold level. Arrows depict the location of primers used for Q-PCR of anti-E2F2 immunoprecipitated chromatin sequences. (**B**) Confirmatory ChIP-Q-PCR analyses of E2F2 target genes in quiescent *E2f2^+/+^* and *E2f2^−^^/^^−^* T lymphocytes (representative experiment of three biological replicates). ChIP assays were performed using anti-E2F2 and anti-T (irrelevant control) antibodies, and Q-PCR was performed using primers spanning genomic regions around or close to E2F consensus motifs in each gene. Data are presented as percentage of input chromatin. The values represent the mean ± SD of triplicate platings. Significant differences in E2F2 enrichment of chromatin in *E2f2^+/+^* versus *E2f2^−^^/^^−^* cells are labeled with an asterisk (*P* < 0.05).

To validate the results of the ChIP-chip analysis, we performed conventional chromatin immunoprecipitation with E2F2 antibodies, followed by quantitative PCR (ChIP-Q-PCR) in lymphocytes obtained from *E2f2^+/+^* and *E2f2^−^^/^^−^* mice. From the ChIP-chip–enriched gene set, we selected six genes known to harbor E2F sites in their promoter (Figure 1A). This set includes *Chk1*, which has been previously shown to bind E2F2 (4), and five other genes related to various aspects of cell cycle regulation (*Mcm5*, *Prkdc*, *Gmnn*, *Polr2a* and *Fancl*). The *β-actin* gene, whose promoter lacks active E2F sites (4), was used as a negative promoter control. Additionally, parallel ChIP assays were carried out with an irrelevant antibody (anti-T) to control for nonspecific chromatin immunoprecipitation. As shown in Figure 1B, E2F2 binding to the promoters of *Chk1*, *Mcm5*, *Prkdc*, *Gmnn*, *Polr2a* and *Fancl*, but not to the promoter of *β-actin*, was significantly enriched in *E2f2^+/+^* cells compared with *E2f2^−^^/^^−^* cells (*P* < 0.05 in all cases).

Gene Ontology analysis revealed that DNA metabolism (e.g. *Rbbp4*, *Parp1*, *Ard1*, *Rpa2*), DNA damage and repair (e.g. *Pms2*, *Ruvbl2*, *Smc1a*, *Uhrh1*, *Fanc1*, *Brca1*, *Chk1*, *ATM*), DNA replication (e.g. *Gmnn*, *Mcm5*, *Cdc6*, *Orc6l*) and mitotic cell cycle (e.g. *Cdc25a*, *Cdc25c*, *Incenp1*, *Ndc80*, *Cdc2*, *Sirt2*) were the most overrepresented biological processes among the 839 genes enriched in the ChIP-chip assay, with enrichment ratios >2 (Table 1 and Supplementary Table S5). Other categories such as RNA processing (e.g. *Dmtf1*, *Hnrnpa1*) and RNA splicing (e.g. *Srsf1*, *Srsf10*, *U2af1l4*) were also overrepresented, albeit with enrichment ratios <2 (Table 1 and Supplementary Table S5).

**Table 1.** Functional classification of E2F2-bound genes, as determined by DAVID

GO term	Genes (N)^a^	Fold enrichment
DNA metabolism	54***	2.1
DNA damage and repair	26***	3
DNA replication	19***	2.9
Mitotic cell cycle	16**	2.15
RNA processing	11*	1.7
RNA splicing	14*	1.8

^a^Number of genes that are included in each GO category (*P < 0.05; **P < 0.01; ***P < 0.0001).

We hypothesized that E2F binding motifs would be overrepresented in the promoters of the E2F2-bound gene set identified by ChIP-chip. To test this hypothesis, we used CEAS (27), a program that determines the number of transcription factor binding motifs within a subset of DNA sequences, and compares the data with the number of copies of each motif present in the entire genome. This analysis showed that the E2F motif was overrepresented in the E2F2-bound DNA sequences in each of the three biological replicates (*P* < 10^−^^10^), with enrichment values of 5, 6 and 8, respectively.

To identify potential E2F motifs in the E2F2-bound promoters, we made use of the MotifLocator tool of the program TOUCAN (28). Using a threshold level of 0.85 for similarity with the canonical E2F motif recorded in the TRANSFAC database (TTTSSCGC), 70% of E2F2-bound genes harbored at least one canonical motif within the −2000/+500 bp regulatory region that was considered for these analyses. This proportion was reduced to 28% when the threshold level was increased to 0.9. This observation suggests that the E2F2 binding motif in most E2F2-bound genes is closely related to the canonical sequence, but it does not match exactly with it. The MotifLocator tool also showed that the majority of these E2F2-binding motifs (73.4%) were close to the transcriptional start site, with a peak frequency located at −100 bp of the initiation site.

### Identification of a set of genes regulated specifically by E2F2 during quiescence

Next, we sought to determine whether the binding of E2F2 to target promoters during quiescence might have an effect on the transcriptional activity of these genes. Using a gene expression microarray, we have previously identified a large set of genes whose expression is deregulated in quiescent *E2f2^−^^/^^−^* lymphocytes (4). By combining these transcriptome data with the data from anti-E2F2 ChIP-chip analysis, we found that the expression of >80% of E2F2-bound genes (including *Polr2a* and *Prkdc*) is not deregulated on E2F2 gene inactivation, suggesting that their transcription is redundantly regulated by other E2F family members. However, we identified a set of 51 E2F2-bound genes whose expression is deregulated (38 upregulated and 13 downregulated) in quiescent *E2f2^−^^/^^−^* lymphocytes (Table 2), suggesting that they are specifically regulated by E2F2. Included in the upregulated subset were *Mcm5*, *Chk1*, *Gmnn* and *Fancl*. The promoters of most genes directly regulated by E2F2 bear E2F binding sites that are also conserved in human and/or rat (Table 2). Moreover, the fraction of genes repressed/activated by E2F2 was substantially higher in the group of E2F2-bound genes (38/13, ratio of 2.92) than in the group of genes not bound by E2F2 (361/192, ratio of 1.88), suggesting that a prominent role of E2F2 in cellular quiescence is to repress in a direct and specific manner the expression of a subset of cell cycle–related genes.

**Table 2.** E2F2-specific target genes and associated GO terms in quiescent T lymphocytes by a combination of ChIP-chip and gene expression microarray

Entrez gene	Gene symbol	E2F motifs^a^	−200 + 200 bp^b^	Up/ down^c^	Entrez gene	Gene symbol	E2F motifs^a^	−200 + 200 bp^b^	Up/ down^c^
DNA replication and metabolism					DNA repair				
23 834	Cdc6	4*	Yes	Up	12 649	Chk1	3*	Yes	Up
57 441	Gmnn	3*	Yes	Up	67 030	Fancl	2*	Yes	Up
17 215	Mcm3	5*	Yes	Up	11 545	Parp1	1*	No	Up
17 218	Mcm5	4*	Yes	Up	18 140	Uhrf1	5*	Yes	Up
17 220	Mcm7	5*	Yes	Up	11 920	Atm	0	No	Down
56 452	Orc6l	3*	Yes	Up					
19 075	Prim1	0	No	Up	Miscellaneous/unknown				
19 891	Rpa2	4*	Yes	Up	54 447	Asah2	1	No	Up
20 133	Rrm1	3*	Yes	Up	12 166	Bmpr1a	5*	Yes	Up
					67 300	Cltc	2*	Yes	Up
Cell cycle regulation					28 040	D6Wsu163e	4*	Yes	Up
108 912	Cdca2	1*	Yes	Up	60 530	Fignl1	4*	Yes	Up
16 319	Incenp	1	Yes	Up	68 537	Mrpl13	7*	Yes	Up
105 837	Mtbp	6*	Yes	Up	70 769	Nolc1	1*	Yes	Up
67 052	Ndc80	1*	Yes	Up	53 893	Nudt5	4*	Yes	Up
52 033	Pbk	0	No	Up	110 809	Sfrs1	2*	No	Up
22 367	Vrk1	2*	No	Up	20 492	Slbp	4*	yes	Up
12 048	Bcl2l1	2	Yes	Down	30 057	Timm8b	1*	No	Up
59 046	Arpp19	1*	No	Up	68 842	Tulp4	2*	Yes	Up
					79 560	Ublcp1	0	No	Up
Nucleobase, nucleotide and nucleoside biosynthesis					66 206	1110059e24Rik	3*	No	Down
66 953	Cdca7	3*	Yes	Up	269 774	Aak1	2*	No	Down
74 838	Narg1	1*	Yes	Up	71 752	Gtf3c2	2*	Yes	Down
54 132	Pdlim1	4*	Yes	Up	101 142	Itfg2	1*	Yes	Down
116 940	Tgs1	0	No	Up	18 807	Pld3	2*	Yes	Down
218 973	Wdhd1	2*	No	Up	19 089	Prkcsh	1*	Yes	Down
26 896	Med14	1*	Yes	Down	68 272	Rbm28	1*	Yes	Down
170 791	Rbm39	3*	Yes	Down	20 462	Sfrs10	2*	Yes	Down
					66 477	Usmg5	4*	No	Down

^a^Number of E2F binding motifs in each gene, identified by TOUCAN within a −2000/+500 bp genomic region. Asterisk denotes that E2F motifs are also present in orthologous gene promoters from human and/or rat, ^b^Indicates whether E2F motifs are located within a −200/+200 bp region in each gene, ^c^Up- or down-regulated in *E2f2^−^^/^^−^* cells (4).

### E2F2 functions as a negative transcriptional regulator of a subset of responsive genes

**Figure 2.**
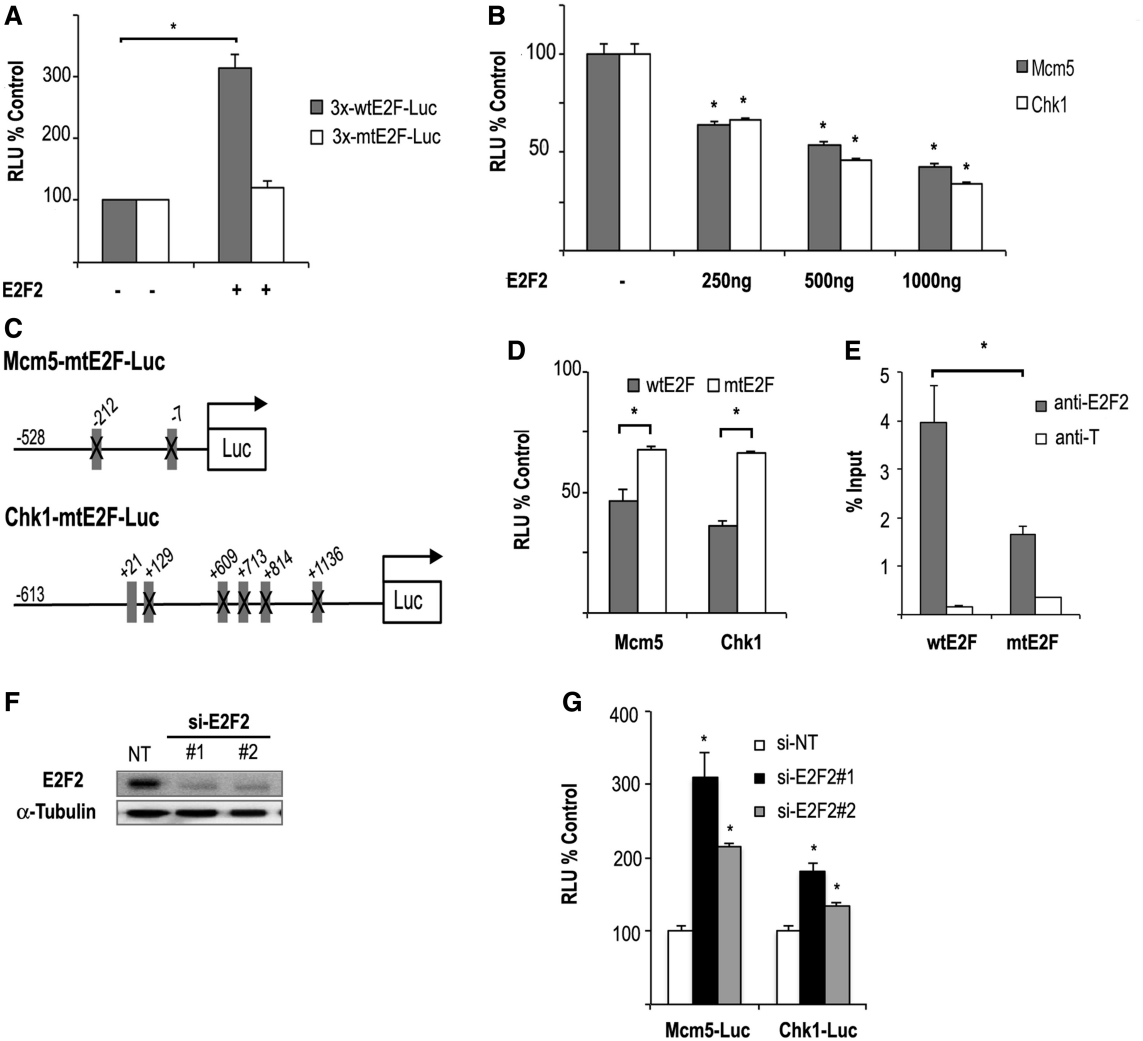
Promoter-dependent transcriptional regulation mediated by E2F2. (**A**) E2F2 activates transcription of a synthetic promoter with three canonical E2F sites (p3X-wt*E2F*-Luc), but not with mutated E2F sites (p3X-mt*E2F*-Luc). HEK293T cells were transfected with the indicated luciferase reporter constructs and 500 ng of pCMVE2F2. A plasmid expressing *Renilla* luciferase was co-transfected to normalize luciferase activity accounting for transfection efficiency. Luciferase activity (RLU) is presented as a ratio of firefly/*Renilla* intensities. Data are shown as percentage over the empty pCMV transfection. The values shown represent the mean ± SD (*n* = 3 independent experiments; *P < 0.05). (**B**) E2F2 represses transcription driven by *Mcm5* and *Chk1* promoters. HEK293T cells were transfected with the indicated luciferase reporter constructs and different concentrations of pCMVE2F2, and analyzed as above. Data are shown as percentage over the empty pCMV transfection (*n* = 4 independent experiments; *P < 0.05). (**C**) Schematic representation of mouse *Mcm5* and human *Chk1* regulatory regions cloned upstream of the luciferase transcriptional unit. Boxes indicate the predicted E2F-binding sites at a 0.9 threshold level; mutated sites in the constructs are crossed out. (**D**) Luciferase assays were performed with pCMVE2F2 (1000 ng) and the indicated WT and mutant reporter constructs. Statistically significant differences were calculated comparing luciferase activity in cells expressing WT promoters versus mutated promoters (*n* = 3 independent experiments; *P < 0.05). (**E**) E2F2 binding to *Mcm5* promoter is dependent on intact E2F sites. HEK293T cells were transfected with pCMVE2F2 and either pMcm5-wtE2F-Luc (wtE2F) or pMcm5-mtE2F-Luc (mtE2F) for 48 h, and ChIPs were performed with anti-E2F2 or with an irrelevant antibody control (anti-T). Immunoprecipitated chromatin was quantified by Q-PCR (see Figure 1 for amplicons). Data are presented as percentage of input chromatin (*P ≤ 0.05). (**F**) Efficient knockdown of E2F2 in U2OS cells. Two independent siRNA molecules for E2F2 or a nontarget siRNA control (si-NT) were transfected into U2OS cells along with *Mcm5* or *Chk1* luciferase reporter constructs. Forty-eight hours after siRNA transfection, protein was recovered and western blots were performed with indicated antibodies. (**G**) Effect of E2F2 knockdown on transcriptional activity of target genes. Luciferase activity driven by *Mcm5* and *Chk1* promoters in U2OS cells transfected with E2F2 siRNA molecules was determined as above. Data are shown as percentage over the NT siRNA transfection. (*n* = 3 independent experiments; *P < 0.05).

The role of E2F2 as transcriptional activator has been previously established (1,2,10). However, little is known about its role in transcriptional repression. The results shown in Table 2 led us to postulate that repression by E2F2 could be causally related to promoter binding by this transcription factor. To test this hypothesis, we examined the effect of E2F2 on gene expression by luciferase assays using reporter constructs carrying E2F consensus motifs. The HEK293T cell line was chosen to carry out these assays because these cells lack detectable expression of endogenous E2F2, and because RB/E2F complexes are disrupted in these cells, owing to the constitutive expression of E1A and large T antigen (30), thus preventing any inhibitory activity of endogenous RB over ectopically expressed E2F2. In agreement with previously published data (31), E2F2 activated the transcription driven by a synthetic promoter containing three adjacent E2F binding sites (3X-wt*E2F*-Luc), but not by a promoter carrying three mutated E2F binding sites (3X-mt*E2F*-Luc) (Figure 2A). By contrast, expression of E2F2 reduced basal *Mcm5* and *Chk1* promoter activity up to ∼40% (*P* < 0.05) compared with cells transfected with empty plasmid (Figure 2B). To examine whether E2F2-mediated repression was dependent on intact E2F sites, we mutated E2F motifs on *Mcm5* and *Chk1* promoters (Figure 2C). In luciferase assays, E2F2-dependent transcriptional repression was significantly reversed when reporter constructs carrying mutations in E2F sites were analyzed (Figure 2D), and, as expected, ChIP analyses showed a considerable reduction of E2F2 binding to the mutated *Mcm5* promoter (Figure 2E). These results indicate that E2F2 can efficiently repress promoters through E2F motifs.

Next, E2F2 expression was knocked down using RNA interference in U2OS cells, which express detectable levels of endogenous E2F2. A substantial reduction of E2F2 expression by two independent siRNA molecules was demonstrated by western blot analysis (Figure 2F). As a control, a nontarget siRNA did not decrease E2F2 expression. Along with the E2F2-specific siRNAs, U2OS cells were transfected with the *Chk1*-Luc or *Mcm5*-Luc reporter constructs, and luciferase reporter assays were carried out. As shown in Figure 2G, the basal luciferase activity of both promoters was increased between 1.3 - and 3-fold after silencing E2F2 (*P* < 0.05), further demonstrating the repressive effect of E2F2 on these target promoters.

**Figure 3.**
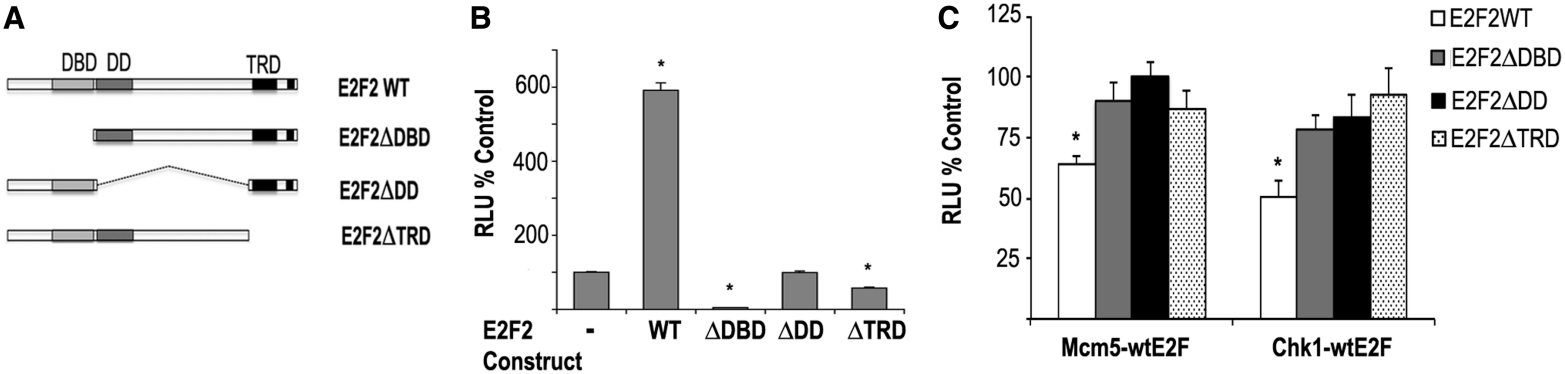
Structural domains involved in activation and repression mediated by E2F2. (**A**) Schematic representation of E2F2 deletion mutant series used for transcription assays. Domains involved in DNA binding (DBD), dimerization (DD) and transactivation/RB-binding (TRD) are shown. (**B** and **C**) Transcriptional activation of 3X-wt*E2F*-Luc and transcriptional repression of *Mcm5*-Luc or *Chk1*-Luc require the DNA binding, dimerization and transactivation domains of E2F2. Expression plasmids of full-length E2F2 (WT) and deletion mutants (ΔDBD, ΔDD, ΔTRD) were introduced into HEK293T cells along with the indicated reporter plasmids. Luciferase activity (RLU) was determined 48 h after transfection. Data are shown as percentage over the samples transfected with empty pCMV (*P < 0.05). The values shown represent the mean ± SD of triplicate platings (representative experiment of two independent experiments).

To elucidate the structural domains of E2F2 involved in transcriptional repression, we examined the effect of several E2F2 deletion mutants on the activity of 3X-*E2F*, *Mcm5* and *Chk1* promoters. Three expression plasmids coding for E2F2 deletion mutants lacking the DNA binding domain (E2F2-ΔDBD), the marked box and dimerization domain (E2F2-ΔDD) or the transactivation and RB binding domain (E2F2-ΔTRD) were generated (Figure 3A). None of the individual E2F2 mutant plasmids was able to activate luciferase activity driven by 3X-wt*E2F*-Luc (Figure 3B), in line with previous observations with E2F1 deletion mutants (32). Importantly, the ability to repress the *Mcm5* and *Chk1* promoters was also lost in all the E2F2 deletion mutants tested (Figure 3C). Of note, luciferase activity remained close to basal on expression of E2F2 deletion mutants when reporter constructs with *Mcm5* or *Chk1* promoters carrying E2F site mutations were tested, thus excluding potential nonspecific effects (Supplementary Figure S1). These results suggest that all three domains of E2F2 are necessary for efficient transcriptional activation as well as for repression of E2F2 target promoters.

### CREB binds to the promoter of a subset of E2F2 target genes, but E2F2 does not regulate CREB expression, phosphorylation or DNA binding activity

It is thought that transcription regulation requires the integration of signals resulting from combinatorial interactions among different transcription factors (33). Consequently, we postulated that E2F2 could cooperate with other transcription factors in the regulation of its target genes. Using the CEAS program, we found that several transcription factor motifs were overrepresented in the set of 839 genes immunoprecipitated by E2F2 in quiescent lymphocytes. In particular, the CRE motif (TGACGT) showed an enrichment ratio of 2. The MotifLocator tool indicated that nearly half (43.59%) of the genes immunoprecipitated by E2F2 exhibit at least one CRE motif within a −2000/+500 bp genomic region at a 0.9 threshold level. In most cases, this motif is located within 300 bp of the transcriptional initiation site, with a peak frequency at −100 bp. Furthermore, a 2.5-fold enrichment of CREB binding sites was also evident in the subset of genes bound to E2F2 and negatively regulated by this factor.

**Figure 4.**
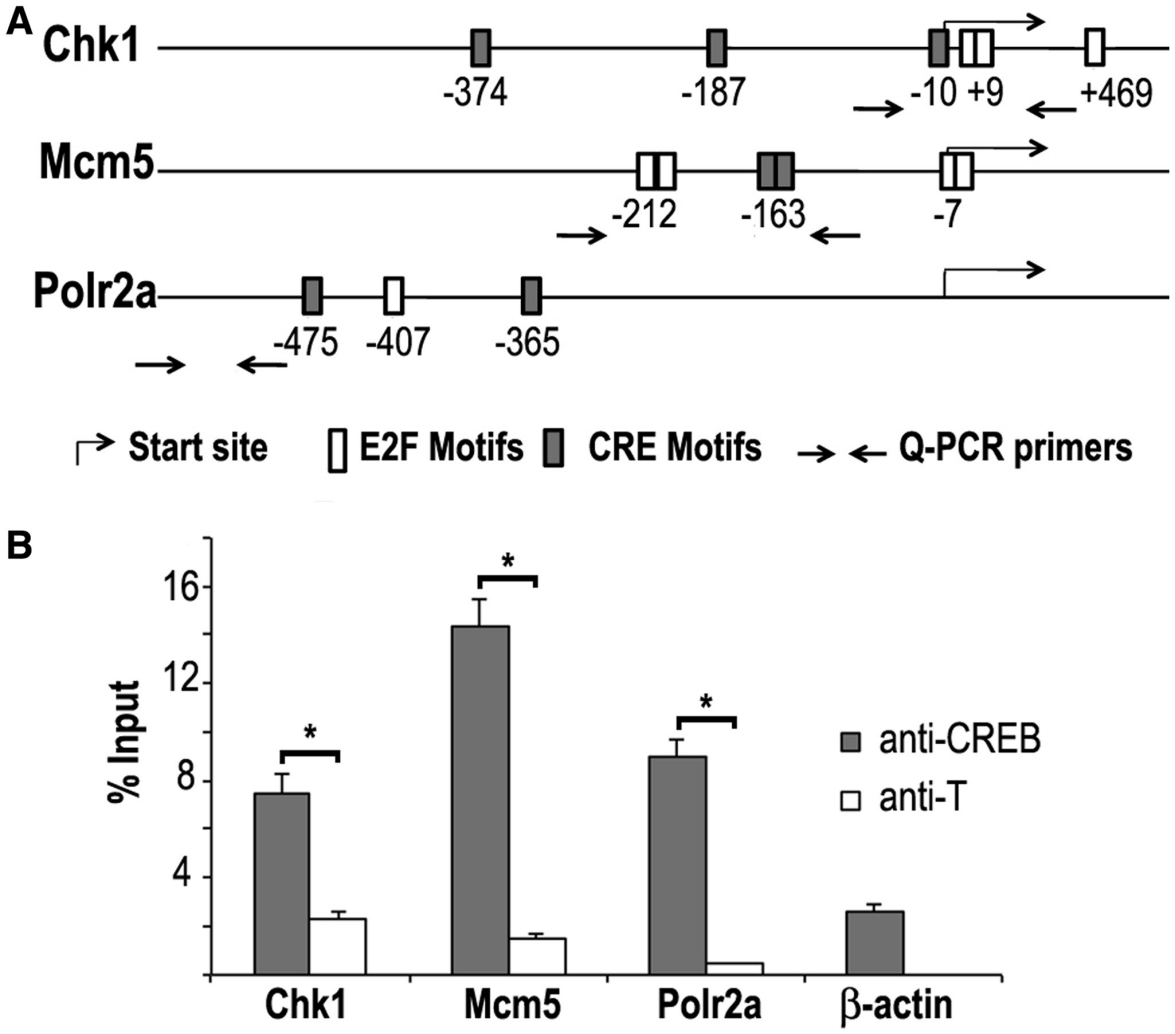
CREB is recruited to the promoter region of a subset of E2F2 target genes. (**A**) Schematic representation of mouse *Chk1*, *Mcm5* and *Polr2a* gene promoter regions, indicating the location of consensus E2F and CRE motifs detected at a 0.9 threshold level. Arrows indicate the location of primers used for Q-PCR of anti-CREB immunoprecipitated chromatin. (**B**) The binding of CREB was assessed by ChIP-Q-PCR using primers spanning genomic regions around or close to E2F and CRE consensus motifs in each gene. ChIP analyses of E2F2 target genes in quiescent T lymphocytes were performed using anti-CREB or anti-T (irrelevant antibody). *β-actin* promoter served as a negative promoter control. Data are presented as percentage of input chromatin. The values represent the mean ± SD (representative experiment of two biological replicates). Significant differences are labeled with an asterisk (*P* < 0.05).

The concomitant enrichment of both E2F and CRE motifs in the promoters that were immunoprecipitated by E2F2 suggested that these motifs could function as cis-regulatory modules. The ModuleSearcher tool of TOUCAN was used to search the promoters of the E2F2-bound gene set for the simultaneous presence of E2F and CRE motifs at a distance of <500 bp from one another. A total of 84 genes (10.57%) exhibited both motifs separated by <500 bp (Supplementary Table S6). Included in this set of genes are *Chk1*, previously shown to be bound by CREB (34) in chromatin immunoprecipitation experiments, as well as *Mcm5*, and *Polr2a* (Figure 4A).

These findings suggest that CREB could be recruited to E2F2 target genes. To test this possibility, we first examined CREB promoter occupancy in E2F2 target genes. Conventional ChIP-Q-PCR assays were performed on chromatin derived from quiescent lymphocytes with antibodies that are specific for CREB. Q-PCR analysis demonstrated robust binding of CREB to the promoter of *Chk1*, *Mcm5* and *Polr2a* (*P* < 0.05), but not to the promoter of *β-actin*, used as negative promoter control (Figure 4B).

**Figure 5.**
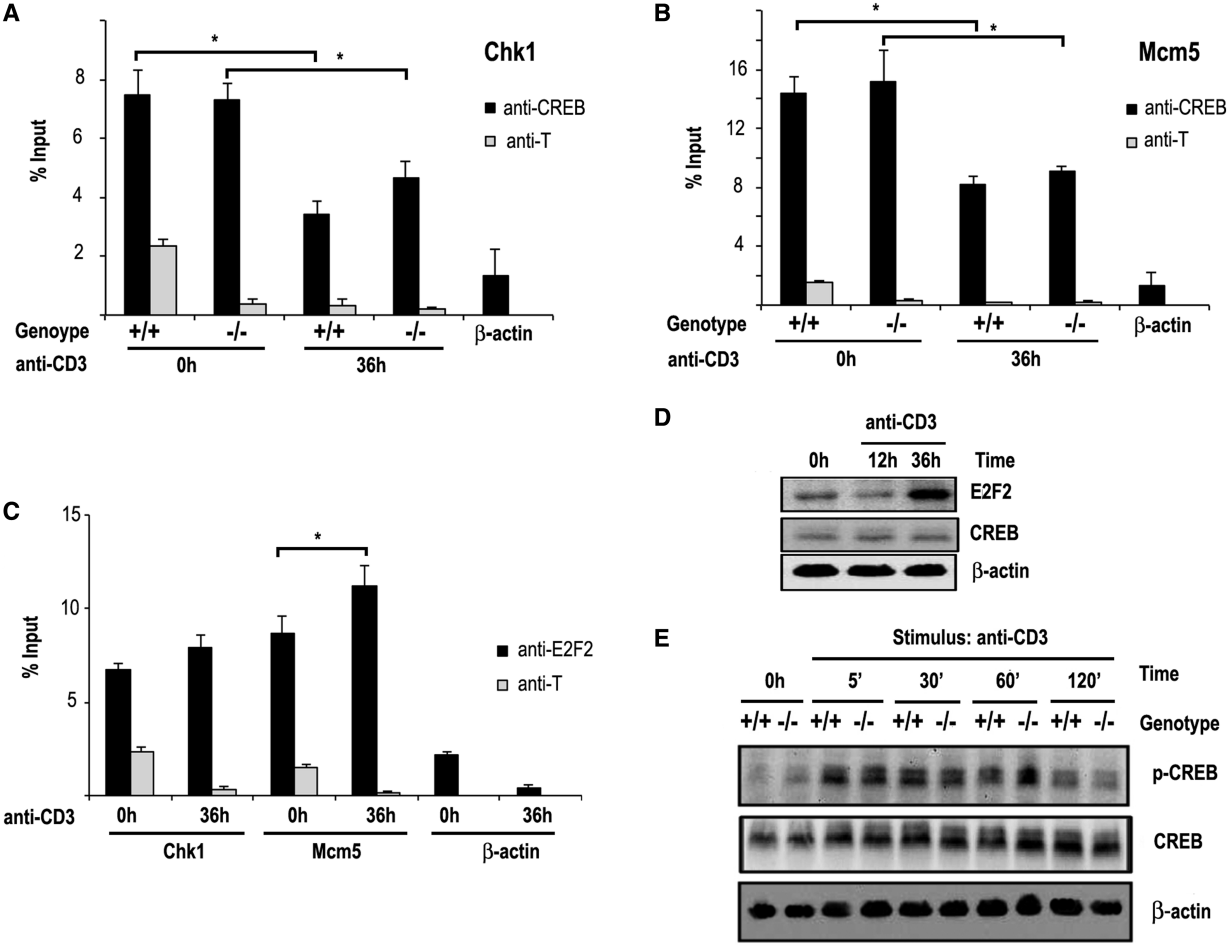
CREB expression, phosphorylation or binding to promoters is E2F2-independent. (**A**, **B**) ChIP analyses of CREB binding to *Chk1* promoter (A) and *Mcm5* promoter (B) in T lymphocytes. Sonicated, cross-linked chromatin of resting (0 h) or anti-CD3 treated (36 h) T lymphocytes derived from *E2f2^+/+^* and *E2f2^−/−^* mice were immunoprecipitated with anti-CREB or an irrelevant antibody control (anti-T), and the purified DNA was analyzed by Q-PCR. Data are presented as percentage of input chromatin. The values represent the mean ± SD (representative experiment of three independent experiments; *P < 0.05). (**C**) ChIP analyses of E2F2 binding to *Chk1* promoter and *Mcm5* promoter in resting (0 h) or anti-CD3 treated (36 h) T lymphocytes derived from *E2f2^+/+^* mice. Data are presented as percentage of input chromatin. The values represent the mean ± SD (representative experiment of three independent experiments; *P < 0.05). (**D**) Expression of CREB and E2F2 in T lymphocytes. Whole-cell extracts were prepared from lymph node–derived *E2f2^+/+^* T cells that had been treated with anti-CD3. The resulting extracts were immunoblotted with specific antibodies against E2F2 and CREB. (**E**) Expression and phosphorylation of CREB in T lymphocytes. Whole-cell extracts were prepared from lymph node–derived *E2f2^+/+^* and *E2f2^−/−^* T cells that had been treated with anti-CD3. The resulting extracts were immunoblotted with specific antibodies against CREB and phospho-CREB (Ser133).

We next examined whether CREB binding to target promoters is regulated by E2F2. For this purpose, ChIP-Q-PCR assays were performed on quiescent *E2f2^+/+^* and *E2f2^−^^/^^−^* T lymphocytes. CREB was present on *Chk1* and *Mcm5* promoters (7% of input and 14% of input, respectively), in both *E2f2^+/+^* and *E2f2^−^^/^^−^* lymphocytes (Figure 5A and B), indicating that occupancy of these promoters by CREB is not affected by the absence of E2F2.

ChIP-Q-PCR assays were also carried out in lymphocytes stimulated with anti-CD3, to determine promoter occupancy of CREB in cells undergoing G1/S progression. In this cell cycle phase, binding of CREB decreased ∼50%, coincident with an increased transcription rate of target genes (1,10), and these results were not affected by the lack of E2F2 (Figure 5A and B). Consistent with previous results (4), we found that binding of E2F2 to target promoters underwent a slight increment after 36 h of anti-CD3 stimulation, which was only significant in the *Mcm5* promoter (Figure 5C). Increased recruitment of E2F2 to promoters coincided with a robust accumulation of newly synthesized E2F2 levels in activated cells (Figure 5D).

No physical interaction between E2F2 and CREB was detected in co-immunoprecipitation experiments (data not shown). Furthermore, western blot analyses demonstrated that neither the expression level nor the phosphorylation kinetics of CREB, which is known to regulate its activity (35), changed in *E2f2^−^^/^^−^* lymphocytes compared with *E2f2^+/+^* controls (Figure 5E), suggesting that CREB expression or phosphorylation are not dependent on E2F2 in T lymphocytes.

### CREB negatively regulates E2F2 target genes

**Figure 6.**
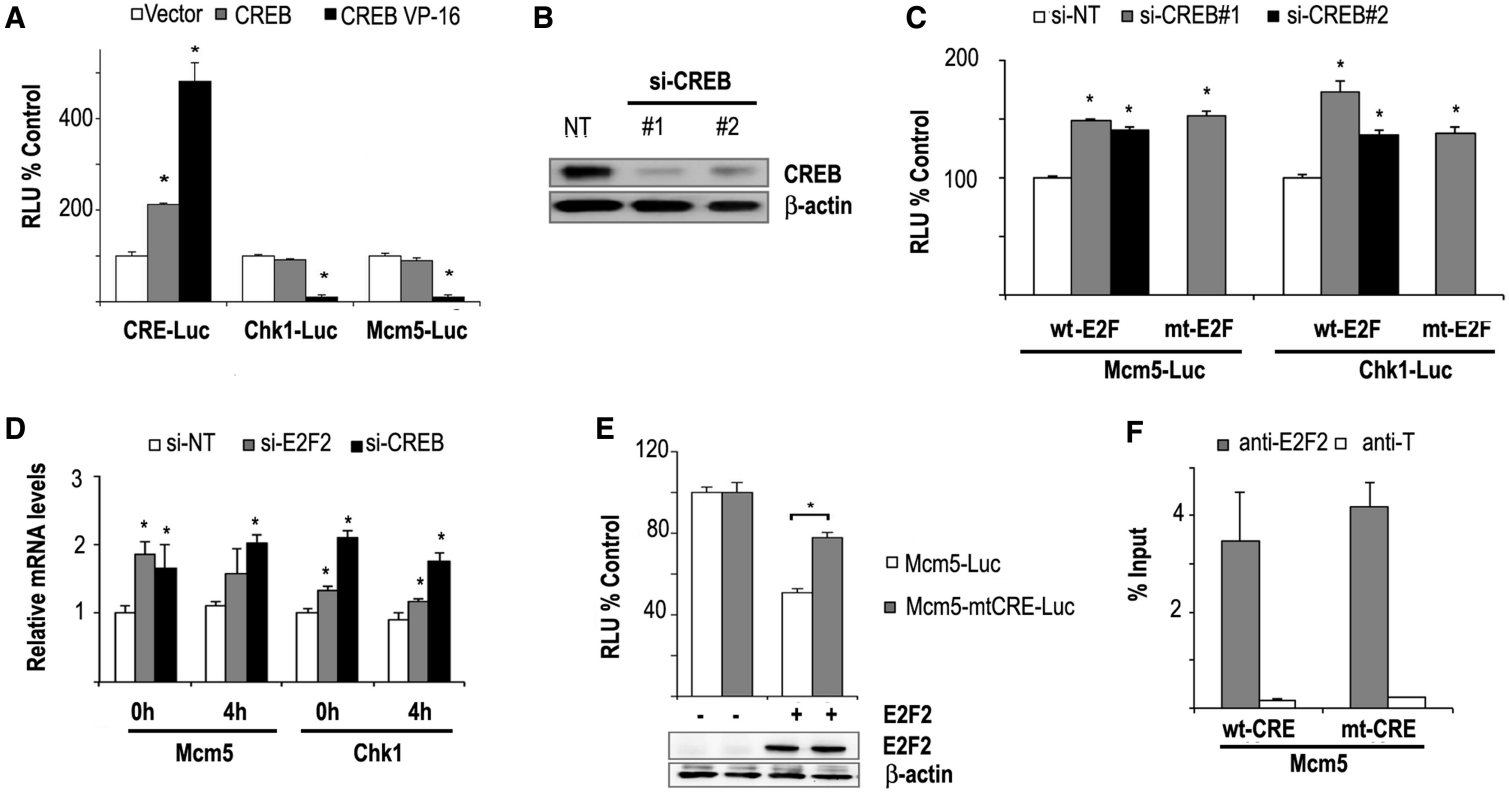
CREB represses E2F target promoters. (**A**) CREB activates the transcription of a reporter construct carrying four CRE motifs, but represses the transcription of *Mcm5* and *Chk1* regulatory regions. Expression plasmids encoding WT CREB or constitutively active CREB (pCREB-VP16) were co-transfected into HEK293T cells with the reporter plasmids pCRE-Luc, p*Chk1*-Luc or p*Mcm5*-Luc, and pRL-TK. Luciferase activity (RLU) is indicated as a ratio of firefly/*Renilla* intensities. Data are shown as percentage over the empty pCMV transfection. The values shown represent the mean ± SD (*n* = 3 independent experiments; *P < 0.05). (**B**) Efficient siRNA-mediated knockdown of CREB. Two independent siRNA molecules for CREB or a nontarget control (si-NT) were transfected into HEK293T cells along with *Mcm5* or *Chk1* luciferase reporter constructs. Forty-eight hours after transfection, protein was recovered and western blots were performed with anti-CREB–specific antibodies. (**C**) Effect of CREB knockdown on the transcriptional activity of target genes. Luciferase activity driven by *Mcm5* and *Chk1* promoters (WT or E2F site mutants) in HEK293T cells transfected with CREB siRNA was determined as above (representative experiment of two independent experiments; *P < 0.05). (**D**) Effect of CREB and E2F2 on mRNA expression of endogenous genes. U2OS cells transfected with CREB or E2F2 siRNA molecules were synchronized in the cell cycle by nocodazole treatment. RT-Q-PCR analyses were carried out with mRNA samples derived from cells at 0 h release or 4 h release from nocodazole treatment. Endogenous expression of *Mcm5* and *Chk1* mRNA was compared with *Hprt* control. Data are represented as fold change relative to 0 h release of nontarget siRNA transfected samples (*P < 0.05). (**E**) Mutation of CRE motifs in Mcm5 promoter partially relieves E2F2-mediated transcriptional repression. E2F2 expression plasmid was co-transfected into HEK293T cells with the reporter plasmids pMcm5-Luc or pMcm5-mtCRE-Luc, bearing mutated CRE motifs. Luciferase activity (RLU) is represented as a ratio of firefly/*Renilla* intensities. Data are shown as percentage over the transfection with the empty pCMV plasmid. The values shown represent the mean ± SD (*n* = 3 independent experiments; *P < 0.05). Western blot shows equal expression of E2F2 in both experimental conditions. (**F**) E2F2 binding to Mcm5 promoter is independent of intact CRE sites. HEK293T cells were transfected with pCMVE2F2 and either p*Mcm5*-wtCRE-Luc (wtCRE) or p*Mcm5*-mtCRE-Luc (mtCRE) for 48 h, and ChIPs were performed with anti-E2F2 or with an irrelevant antibody control (anti-T). Immunoprecipitated chromatin was quantified by Q-PCR. Data are presented as percentage of input chromatin. The values represent the mean ± SD of triplicate platings.

We next analyzed the effect of CREB expression on the activity of target luciferase reporter plasmids in HEK293T cells. As expected, ectopic expression of WT CREB activated the transcription of a synthetic reporter construct with four CRE motifs (CRE-Luc). Additionally, overexpression of a constitutively active mutant form of CREB (CREB-VP16) in which the full-length CREB protein is fused to the transactivation domain of the viral transcriptional coactivator VP16, further increased the activity of the synthetic promoter harboring four CRE motifs (Figure 6A), as previously reported (35). On the other hand, although expression of WT CREB did not affect luciferase expression driven by *Mcm5* and *Chk1*, constitutively active CREB did significantly reduce basal *Chk1* or *Mcm5* promoter activity (Figure 6A). Furthermore, knockdown of endogenous CREB by two independent siRNA molecules significantly increased the basal transcriptional activity of *Chk1* and *Mcm5* promoters (*P* < 0.05 in all cases) in HEK293T cells (Figure 6B and C). Luciferase activity of *Mcm5* or *Chk1* reporter constructs carrying E2F site mutations was similarly increased relative to basal levels upon siRNA knockdown (Figure 6C), suggesting that CREB function is independent of E2F binding activity.

To demonstrate negative regulation by CREB in a more physiological setting, we examined endogenous gene expression after CREB knockdown. For this purpose, U2OS cells were transfected with CREB siRNAs, and synchronized in the cell cycle after treatment with nocodazole followed by release in fresh media. Remarkably, knockdown of CREB by RNAi led to increased expression of endogenous *Mcm5* and *Chk1* mRNA levels (Figure 6D). Upregulation of target genes was not accompanied by accelerated S-phase entry, and cell cycle distribution after CREB depletion was similar to nontarget siRNA-treated cells (Supplementary Figure S2). As expected, expression of these target genes was also higher in cells depleted of E2F2 by RNA interference (Figure 6D).

To test the possibility that CREB DNA binding activity contributes to E2F2-dependent transcriptional repression, we mutated the two overlapping CRE motifs located on *Mcm5* promoter to disrupt CREB binding. As shown in Figure 6E, mutation of these motifs partially relieved E2F2-mediated transcriptional repression of Mcm5 (*P* < 0.05). ChIP experiments revealed that E2F2 recruitment to *Mcm5* promoter was not significantly altered on disruption of CREB binding sites compared with recruitment to WT *Mcm5* promoter (Figure 6F), suggesting that E2F2 binding to its promoters does not depend on intact CRE motifs.

### CREB cooperates with E2F2 in the transcriptional regulation of E2F2 target genes

**Figure 7.**
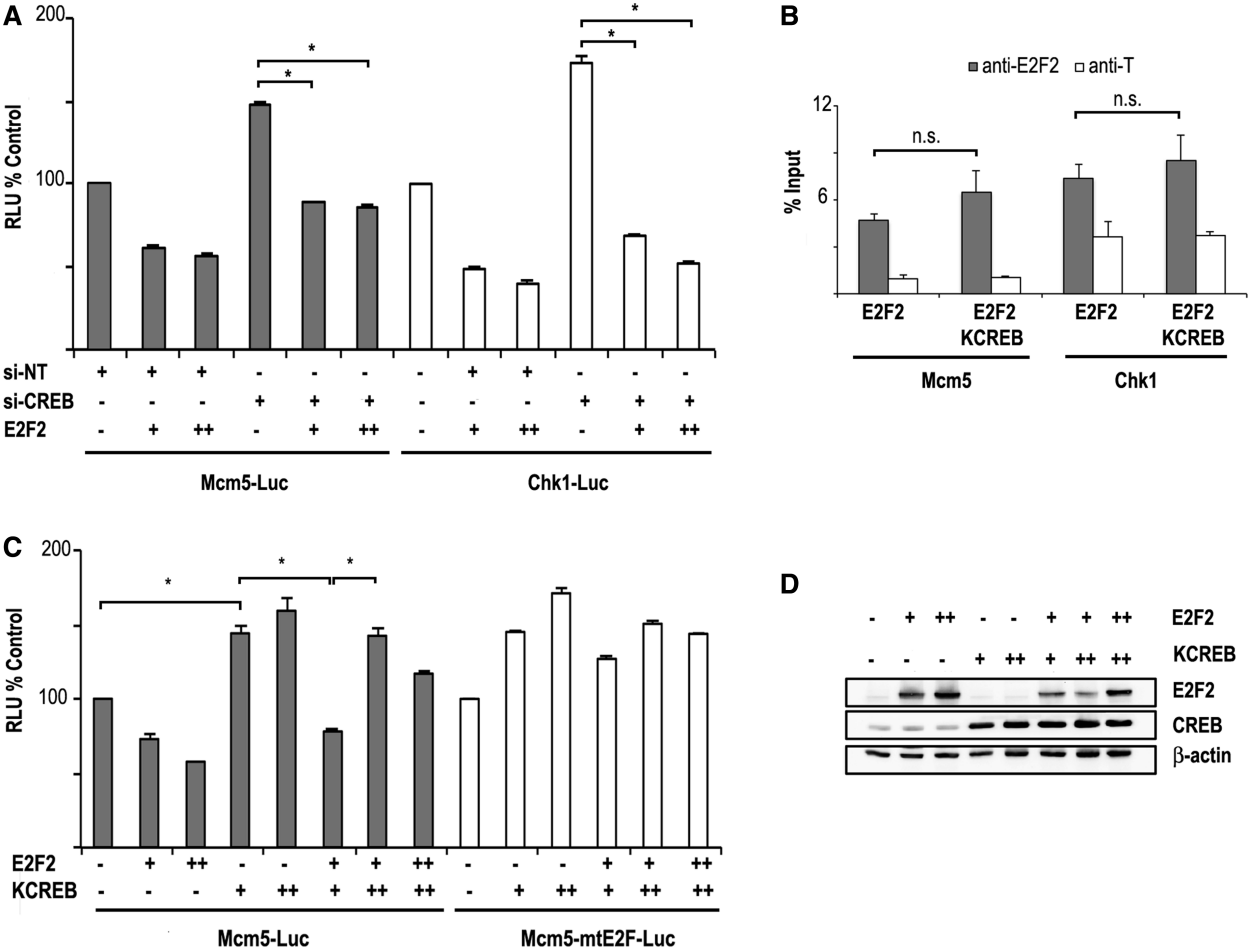
CREB and E2F2 cooperate in transcriptional repression. (**A**) E2F2 enhances CREB-dependent transcriptional repression. Luciferase activity driven by *Mcm5* and *Chk1* promoters was determined in HEK293T cells transfected with CREB siRNA along with pCMVE2F2 (for p*Mcm5*-Luc, + = 250 ng; ++ = 500 ng; for p*Chk1*-Luc, + = 500 ng; ++ = 1000 ng). Data are shown as percentage over the samples transfected with empty pCMV (representative experiment of two independent experiments; *P < 0.05). (**B**) E2F2 binding to endogenous *Mcm5* or *Chk1* promoters is not significantly altered by overexpression of dominant negative KCREB. HEK293T cells were transfected with expression constructs of E2F2 and KCREB for 48 h, and ChIPs were performed with anti-E2F2 or with an irrelevant antibody control (anti-T). Immunoprecipitated chromatin was quantified by Q-PCR (see Supplementary Table S3 for primer sequences). Data are presented as percentage of input chromatin. The values represent the mean ± SD of triplicate platings. (**C**) Expression plasmids of dominant-negative mutant CREB and E2F2 ( + = 250 ng; ++ = 500 ng) were introduced into HEK293T cells along with the reporter plasmids p*Mcm5*-Luc or p*Mcm5*-mtE2FLuc. Luciferase activity is represented as a ratio of firefly/*Renilla* intensities. Data are shown as percentage over the transfection with the empty pCMV plasmid (representative experiment of two independent experiments; *P < 0.05). (**D**) Western blot shows expression of E2F2 and KCREB in the samples used for luciferase assay.

The lack of a detectable E2F2/CREB physical interaction does not necessarily rule out the possibility that E2F2 and CREB could co-regulate target gene expression. To evaluate this possibility, we examined the effect of E2F2 overexpression on luciferase reporter activity in HEK293T cells depleted of CREB by RNA interference. Figure 7A shows that the increased transcriptional activity exhibited by *Mcm5* or *Chk1* promoters after knockdown of CREB was curbed after E2F2 expression.

To verify these results, we made use of KCREB, a dominant negative construct of CREB carrying a mutated DNA binding domain that abrogates CREB-mediated transcriptional activity (36). First, we confirmed that expression of E2F2 and KCREB did not affect E2F2 binding activity to endogenous *Chk1* or *Mcm5* promoters (Figure 7B), suggesting that E2F2 binding to its promoters is not CREB dependent. Additionally, we checked that expression of E2F2, KCREB or both did not alter significantly the cell cycle distribution profile of HEK293T cells (Supplementary Figure S3). In luciferase assays, transfection of KCREB into HEK293T cells increased significantly the basal transcriptional activity driven by *Mcm5* promoter, and abrogated E2F2-mediated transcriptional repression (Figure 7C). Conversely, the increased transcriptional activity of *Mcm5* promoter on overexpression of KCREB could be neutralized by E2F2 (Figure 7C). As a control, western blots showed correct expression of E2F2 and KCREB in the samples (Figure 7D).

Altogether, these findings show that CREB binding motifs contribute to the negative regulation of E2F2 target gene promoters, suggesting that E2F2 and CREB may cooperatively regulate promoters carrying an E2F-CREB regulatory module.

## DISCUSSION

In this work, we have focused on the mechanism by which E2F2 regulates gene transcription. Our results indicate that E2F2 has a widespread role as a negative regulator of gene expression. Using genome-wide assays for transcription factor binding (ChIP-chip) in combination with expression profiling and classical biochemical approaches, we have uncovered a novel partner in the regulation of E2F2-mediated transcriptional repression. We show that E2F2 and CREB are recruited to a subset of E2F target genes to co-regulate their expression at the transcriptional level, providing new insights into E2F regulatory complexity.

Our data indicate that E2F2 is predominantly recruited to the proximal promoter (<1 kb from the transcriptional start site) of both positively and negatively regulated target genes, similarly to E2F1, E2F4 and E2F7 (8,37,38). Furthermore, as observed for other E2Fs (8,38,39), the majority of E2F2 target promoters do not harbor the exact TTTSSCGC motif identified *in vitro* (40), but motifs that are similar to this consensus. E2F2 shares a number of transcriptional targets with other E2F family members *in vivo* (Supplementary Figure S4), supporting the notion of functional redundancy in the family. The E2F2-targeted set is enriched in genes related to DNA replication and repair, DNA damage response, mitosis and RNA processing, suggesting that E2F2 is primarily involved in cell growth control in T lymphocytes. Interestingly, genes belonging to these functional categories are also recruited by other E2F members, and may thus constitute a set of ‘classical’ E2F targets. Many other E2F2-bound genes are involved in a wide variety of functions, such as sensory perception, cognition or response to stress, among others, although no enriched functional categories were observed in our analysis. Further ChIP validation assays performed in a diversity of cell types should provide a more comprehensive list of genes that can recruit E2F2 to their promoters, and may help define additional E2F2-regulated cell functions.

The capacity of E2Fs, including E2F2, to activate transcription has been shown in numerous studies using synthetic promoters carrying E2F sites to drive the expression of the luciferase reporter gene (18,32). However, little is known on the mechanism by which E2F2 can suppress transcription. Our data indicate that E2F2 can repress transcription in cellular contexts harboring active RB (quiescent lymphocytes), but also inactive RB (HEK293T and U2OS cells), suggesting both RB-dependent and RB-independent mechanisms of transcriptional control mediated by E2F2. Importantly, E2F2 mediates repression primarily through E2F motifs. Mutations in two E2F motifs in *Mcm5* promoter that exhibit high similarity to the canonical site (threshold of 0.9) led to a significant, albeit not complete, reversal of E2F2-mediated repression of luciferase activity. Of note, a search for E2F sites at a lower threshold level (0.85) revealed the existence of two additional putative E2F sites in *Mcm5* promoter. These less-similar E2F sites might account for the residual binding and repressive activity that we find with the *Mcm5* promoter mutated in the two canonical E2F sites (Figure 2D and E). Thus, E2F2-mediated repression is likely to be mediated largely through E2F sites.

E2F3b and E2F4 are also known to repress transcription by RB-independent mechanisms in some contexts. E2F3b represses Arf expression in MEFs, and attenuates gene transcription in myoblasts and myotubes in the absence of RB (12,41). Similarly, transcriptional repression of a subset of E2F4 targets does not depend on RB in mouse embryonic fibroblasts and HeLa cells (13). In all these cases, it has been suggested that gene repression could occur through interaction of E2Fs with specific proteins, such as HCF-1 (13). In the case of E2F2, HCF-1 has been shown to interact with several factors, including the YY1–RYBP protein complex and p110 CUX1 on the promoters of *cdc6* and *Polα*, respectively (16,17). However, the functional consequence of these interactions has been to promote transcriptional activation, but not repression, and no E2F2 corepressors have been identified to date.

Our work has revealed that as many as 10% of the genes that bind E2F2 also harbor putative cis-regulatory elements composed of E2F and CREB binding motifs. Furthermore, we show that both E2F2 and CREB are bound to these motifs in quiescent T lymphocytes, coinciding with transcriptional silencing of target genes involved in DNA replication and cell cycle regulation. During G1/S transition, E2F2 binding to target promoters remains unchanged or is slightly increased, whereas CREB binding is diminished. We still do not know how these variations of E2F2 and CREB binding activity are related to gene expression. Although we initially hypothesized that E2F2 could interact physically with CREB, co-immunoprecipitation experiments did not reveal such interaction. Furthermore, lack of binding of E2F2 to target promoters in *E2f2^−^*^/−^ cells did not influence CREB binding to these promoters, and conversely, E2F2 recruitment was not dependent on CREB binding activity. Nevertheless, these results do not preclude the possibility of a weak or transient physical interaction between E2F2 and CREB, or the existence of other factors that may connect both proteins on binding to their specific DNA motifs, and thereby regulate transcription.

The role of CREB on lymphocyte proliferation remains controversial. Early work in transgenic mice that express a dominant-negative form of CREB showed that thymocytes and T cells display a proliferative defect characterized by decreased IL-2 levels, G1 cell cycle arrest and apoptosis, suggesting that CREB is required for cell cycle progression of T lymphocytes (42). However, subsequent work in mice carrying inactivating mutations of *Creb* has not been able to confirm these results. *Creb* knockout mice display reduction in thymic cellularity only after concomitant inactivation of *Atf1*, and lymphocyte proliferation is unaltered in these mice (43). In agreement with these results, we find that CREB is dispensable for cell cycle progression in U2OS cells, even though it appears to play a role in the repression of cell cycle regulatory genes, such as *Mcm5* or *Chk1*. These results imply the existence of a set of genes required for cellular proliferation that are not under CREB control.

Earlier work had already reported enrichment in CRE motifs among E2F target genes, but the functional relevance of this finding was unknown (34,44,45). Functional classification of the genes carrying E2F/CRE cis-regulatory elements suggests that E2F2 and CREB cooperate in the transcriptional repression of genes involved in DNA metabolism and cell cycle control. CREB was originally identified as a transcription factor involved in cAMP-mediated responses. According to the classical model, CREB phosphorylation by PKA in Ser133 leads to the recruitment of the transcriptional activator CBP to the promoters of its target genes, and results in transcriptional activation (35). However, subsequent studies have unveiled the complexity of CREB-mediated gene regulation. Many signaling pathways have an impact on the functional activity of CREB, which may, in turn, activate or repress transcription of its target genes, notably those involved in transcription, synapsis, signaling, metabolism, survival, stress and cellular proliferation (34). In fact, the role of CREB in transcriptional repression is being increasingly appreciated. It has been shown, for example, that constitutively active CREB inhibits the expression of cell cycle–regulating genes in smooth muscle cells, promoting a reduction of their proliferation rates (46). CREB has also been shown to repress *IL-2*, *c-jun* or *loricrin* expression through the arrangement of repressive heterodimers with other CREB family members such as CREM (47–49). Our ChIP and luciferase assay results provide several lines of evidence suggesting that CREB participates in the transcriptional silencing of a subset of E2F2 gene targets. Firstly, CREB is bound to E2F target genes in G0, and its binding activity diminishes significantly during G1/S, coinciding with an increase in the expression of these genes. Secondly, mutation of the CRE motif results in partial rescue of E2F2-mediated repression. Moreover, silencing of CREB or blocking of its DNA binding activity leads to an increase in transcriptional activity of target genes. Interestingly, cooperative repression by E2F1 and CREB has been recently demonstrated in the transcriptional regulation of AP-2a, a tumor suppressor gene involved in the malignant phenotype of melanoma, whose promoter harbors three CRE-like sites and one E2F site necessary for its regulation (50). This finding parallels the cooperative repression of *Mcm5* and *Chk1* promoters by E2F2 and CREB described here. It is likely that future work will uncover further examples of genes that are regulated coordinately by E2F members and CREB.

Taken together, our data reveal a novel layer of complexity in the regulation of E2F-responsive genes. We have shown that E2F2 functions not only as a transcriptional activator but also as a repressor. We have identified a novel molecular mechanism by which two oncogenic transcription factors, E2F2 and CREB, cooperate to negatively regulate a subset of genes involved in the processes of DNA metabolism and cell cycle control. Identifying the components of this novel repressor complex will be the subject of future investigations.

## SUPPLEMENTARY DATA

Supplementary Data are available at NAR Online.

## FUNDING

Spanish Ministry of Economy and Competitiveness [CSD2007-00017, SAF2012-33551 to A.M.Z.]; Basque Government [Etortek-IE12-331, IT634-13 to A.M.Z.]; University of the Basque Country [UFI11/20 to A.M.Z.]. Funding for open access: University of the Basque Country [UFI11/20].

*Conflict of interest statement*. None declared.

## Supplementary Material

Supplementary Data
