# Supplementary material for: E2F2 and CREB cooperatively regulate transcriptional activity of cell cycle genes
Source: Nucleic Acids Res. 2013 Sep 12;41(22):10185–98. doi: 10.1093/nar/gkt821 (PMC3905855; doi:10.1093/nar/gkt821)
Supplement: Supplementary Data [file supp_41_22_10185__index.html]

E2F2 and CREB cooperatively regulate transcriptional activity of cell cycle genes — E2F2 and CREB cooperatively regulate transcriptional activity of cell cycle genes — Supplementary Data 

# E2F2 and CREB cooperatively regulate transcriptional activity of cell cycle genes

## Supplementary Data

files

**Files in this Data Supplement:**

- Supplementary Data - pdf file
- Supplementary Data - xls file
