# Supplementary material for: E2F2 and CREB cooperatively regulate transcriptional activity of cell cycle genes
Source: Nucleic Acids Res. 2013 Sep 12;41(22):10185–98. doi: 10.1093/nar/gkt821 (PMC3905855; doi:10.1093/nar/gkt821)

Supplementary information

To accompany Laresgoiti et al., <u>"E2F2 and CREB cooperatively regulate transcriptional activity of cell cycle genes"</u>

<u>Figure Legends</u>

Supplementary Figure 1. Transcriptional repression of *Mcm5* or *Chk1* requires the DNA binding, dimerization and transactivation domains of E2F2, and intact E2F binding motifs in the responsive promoters. Expression plasmids of full-length E2F2 (WT) and deletion mutants (ΔDBD, ΔDD, ΔTRD) were introduced into HEK293T cells along with pMcm5-mtE2F or pChk1-mtE2F. Luciferase activity (RLU) was determined 48h after transfection. Data are shown as percentage over the samples transfected with empty pCMV. The values shown represent the mean ± S.D. of triplicate platings (representative experiment of two independent experiments).

Supplementary Figure 2. Cell cycle progression is independent of CREB**.** U2OS cultures transiently transfected with siRNA molecules targeting E2F2 or CREB were incubated with thymidine followed with nocodazole to synchronize cells in the cell cycle. Cells at G2/M phase were collected by mitotic shake off (0h release), or seeded for entry into the cell cycle (3h release). Cell cycle distribution was assessed by flow cytometry.

Supplementary Figure 3. Cell cycle progression is unaltered by ectopic expression of E2F2 or KCREB. HEK293T cells were transiently transfected with E2F2 and/or KCREB expression plasmids (500ng of each plasmid). Cells were stained with propidium iodide and cell cycle distribution was analyzed by flow cytometry.

Supplementary Figure 4. A Venn diagram depicting the number of genes bound by individual E2F transcription factors: E2F2 (red), E2F1 (green), E2F4 (yellow) and E2F6 (blue). Intermediate colors represent groups of genes that are shared between two E2F members. E2F1, E2F4 and E2F6 target gene data are taken from Xu, X.Q. *et al.*, (37).

References

37. Xu,X.Q., Bieda,M., Jin,V.X., Rabinovich,A., Oberley,M.J., Green,R. and Farnham,P.J. (2007) A comprehensive chip-chip analysis of E2F1, E2F4, and E2F6 in normal and tumor cells reveals interchangeable roles of E2F family members. *Genome Res.*, **17**, 1550–1561.

Supplementary Figure S1

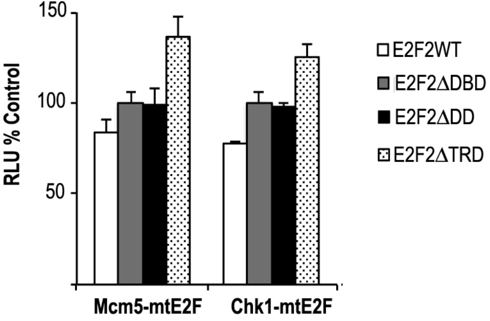

Supplementary Figure S2

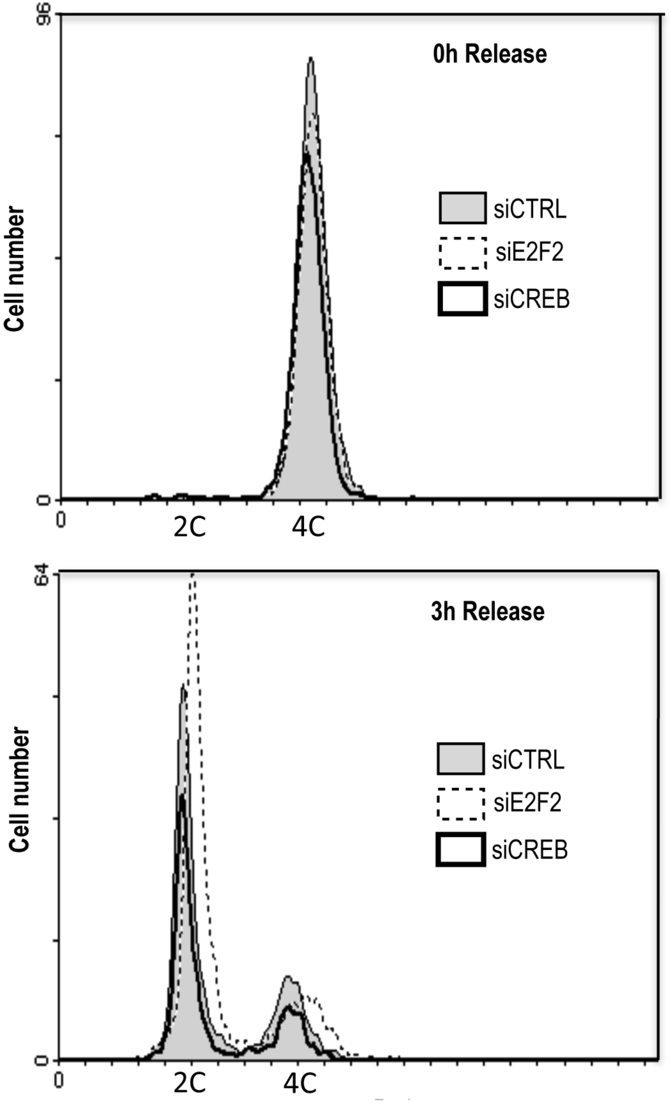

Supplementary Figure S3

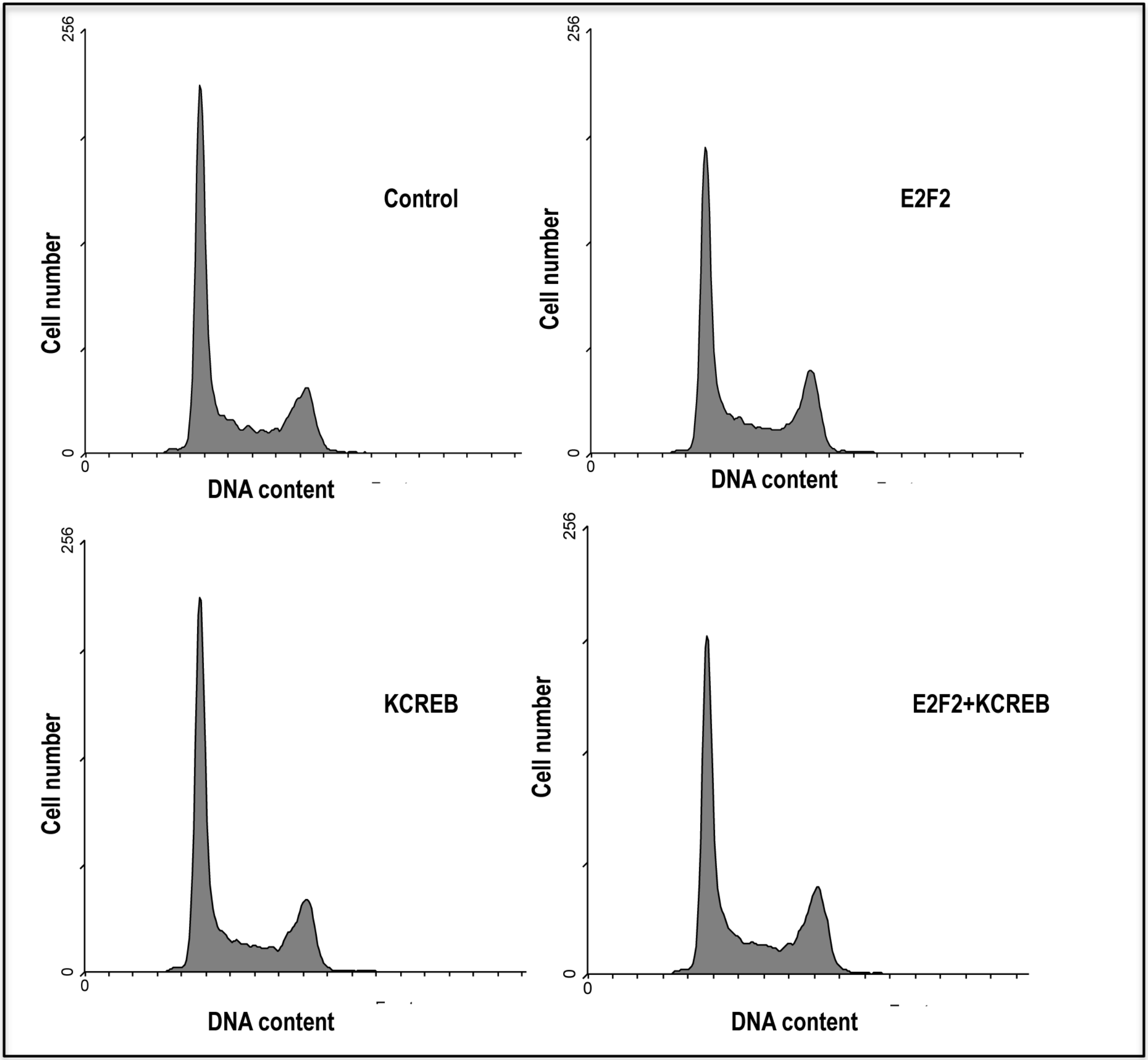

Supplementary Figure S4

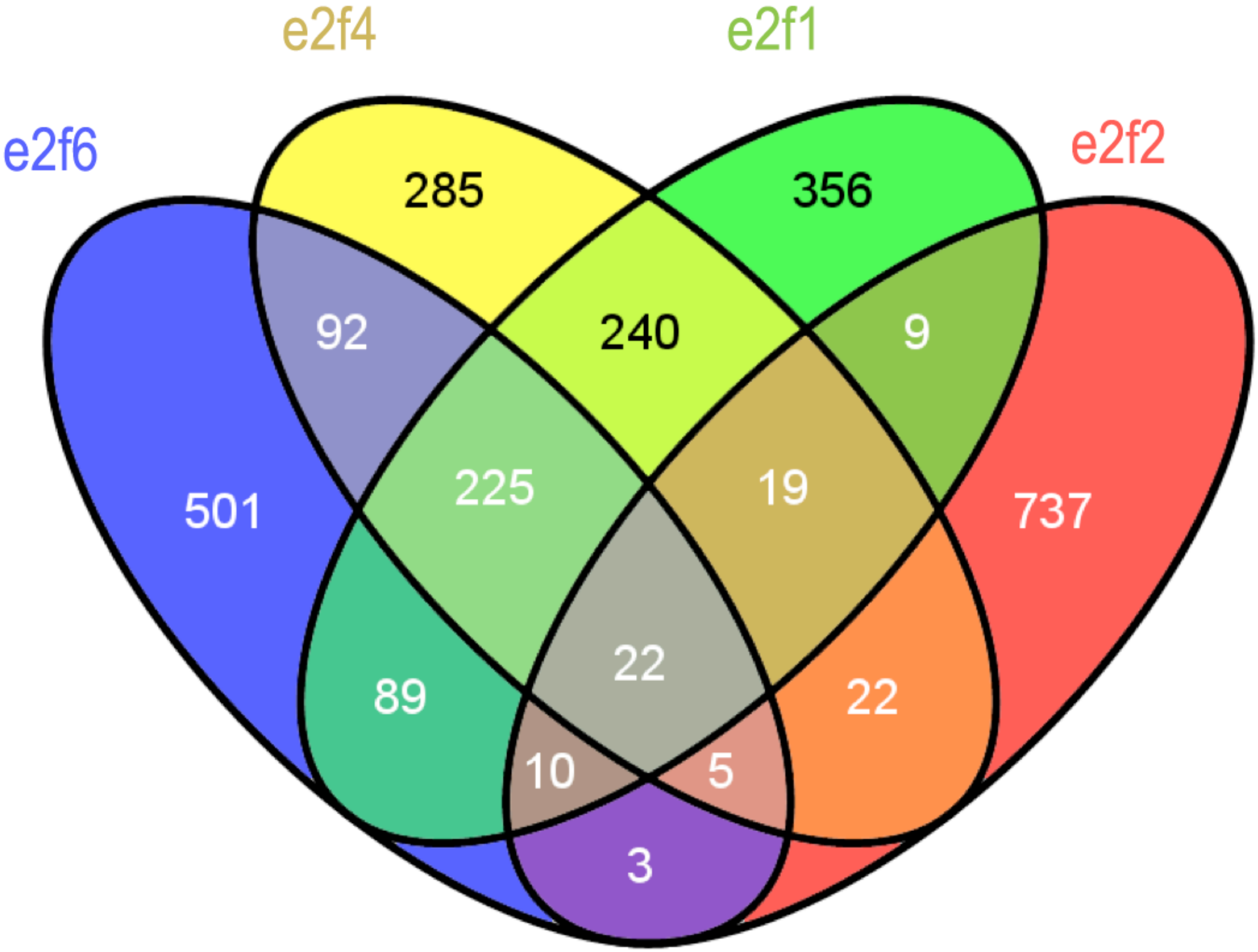

Supplement: Supplementary Data [file supp_gkt821_nar-01879-x-2013-File009.pdf]
